# Secondary-structure prediction revisited: Theoretical β-sheet propensity and coil propensity represent structures of amyloids and aid in elucidating phenomena involved in interspecies transmission of prions

**DOI:** 10.1371/journal.pone.0171974

**Published:** 2017-02-15

**Authors:** Yuzuru Taguchi, Noriyuki Nishida

**Affiliations:** Division of Cellular and Molecular Biology, Department of Molecular Microbiology and Immunology, Nagasaki University Graduate School of Biomedical Sciences, Nagasaki, JAPAN; Deutsches Zentrum fur Neurodegenerative Erkrankungen, GERMANY

## Abstract

Prions are unique infectious agents, consisting solely of abnormally-folded prion protein (PrP^Sc^). However, they possess virus-like features, including strain diversity, the ability to adapt to new hosts and to be altered evolutionarily. Because prions lack genetic material (DNA and RNA), these biological phenomena have been attributed to the structural properties of PrP^Sc^. Therefore, many structural models of the structure of PrP^Sc^ have been proposed based on the limited structural information available, regardless of the incompatibility with high-resolution structural analysis. Recently hypothesized models consist solely of β-sheets and intervening loops/kinks; i.e. parallel in-register β-sheet and β-solenoid models. Owing to the relative simplicity of these structural models of PrP^Sc^, we hypothesized that numerical conversion of the primary structures with a relevant algorithm would enable quantitative comparison between PrPs of distinct primary structures. We therefore used the theoretical values of β-sheet (Pβ) and random-coil (Pc) propensity calculated by secondary structure prediction with a neural network, to analyze interspecies transmission of prions. By reviewing experiments in the literature, we ascertained the biological relevance of Pβ and Pc and found that these classical parameters surprisingly carry substantial information of amyloid structures. We also demonstrated how these parameters could aid in interpreting and explaining phenomena in interspecies transmissions. Our approach can lead to the development of a versatile tool for investigating not only prions but also other amyloids.

## Introduction

Many incurable neurodegenerative diseases, including Alzheimer's disease, Parkinson's disease and tauopathy, are caused by misfolding and amyloid formation of constitutively-expressed proteins. Although these amyloidogenic proteins physiologically exist as monomers in normal conformations, their misfolding results in the formation of amyloid nuclei/fibers. These nuclei, in turn, induce misfolding of normal conformers, incorporating these misfolded proteins into amyloids, a process referred to as seeding. This growth in the size and number of amyloids eventually causes diseases.

Transmissible spongiform encephalopathies (TSEs) are also a group of neurodegenerative diseases caused by misfolding of prion protein (PrP), which include Creutzfeldt-Jakob disease (CJD) in humans, bovine spongiform encephalopathies (BSE) in cattle, chronic wasting disease (CWD) in elk and scrapie in sheep. Unlike the propagation of other amyloids, the propagation of misfolded PrP (PrP^Sc^) in TSEs is so efficient that it practically behaves as an infectious agent called prion. Prions are very similar to viruses, despite lacking genetic material such as DNA or RNA. For example, they propagate efficiently in their preferred host species, e.g. BSE prefers cattle and sheep, whereas transmission to other species is often inefficient with longer incubation periods, i.e. species barrier. However, once the barrier is overcome, the host range of the prion changes and secondary passage to other individuals in that species is more efficient with shorter incubation times, i.e. adaptation [1]. Moreover, in transmission of TSEs, the clinicopathological traits of the donor, including clinical course, distribution of lesions and PrP deposition patterns, are faithfully reproduced in the recipient, resulting in prions being mistaken for viruses. Thus, each prion with unique clinicopathological properties was called a “strain”.

The protein-only hypothesis states that the structures of PrP^Sc^ contain strain-specific pathogenic information and that this information is inherited when the host-encoded normal conformer PrP (PrP^C^) is refolded by PrP^Sc^ in template-directed manner [1]. Despite the importance of PrP^Sc^ structures, however, they remain undetermined owing to their incompatibility with conventional high-resolution structural analysis. Nevertheless, structural information on PrP^Sc^ has been collected and structural models were formulated through methods such as electron microscopic analysis of scrapie fibrils [2], hydrogen/deuterium-exchange mass spectrometry [3], disulfide cross-linkage of *in vitro*-formed PrP fibrils [4] and *in silico* analysis [5, 6]. Although previous models contained α-helices [2, 4, 5], more recent models devoid of α-helices, including the β-solenoid [7] and parallel in-register intermolecular β-sheet [8–10] models, are thought to be more plausible. To date, however, none of these models has explained the mechanisms underlying the virus-like properties of prions.

If PrP^Sc^ consists solely of β-sheets and intervening loops/kinks, then PrP^C^-PrP^Sc^ interactions would be relatively simple, especially in parallel in-register models, where each region of the substrate PrP^C^ interacts with the corresponding region of the template PrP^Sc^. This simple interaction modality suggested that numerical conversion of the primary structure of PrPs by algorithms that can render structural information of PrP^Sc^ to the converted values would enable PrPs of different primary structures to be quantitatively compared. This, in turn, would facilitate interpretation of results of interspecies transmission experiments, and moreover, arithmetic operations on the converted values might reflect certain aspects of PrP^C^-PrP^Sc^ interactions. Therefore, we attempted to use theoretical propensity to β-sheet (Pβ) formation, calculated by a neural-network secondary structure prediction (http://cib.cf.ocha.ac.jp/bitool/MIX/) [11], based on the following four assumptions: (i) PrP^Sc^ consists only of β-sheets and intervening loops/kinks; (ii) the regional structures of PrP^Sc^, including positions of the β-strands, are determined by 'actual' intrinsic propensity to β-sheet formation; (iii) because PrP^Sc^ contains no α-helical regions, the theoretical propensity to α-helix (Pα) formation can be ignored; and (iv) the Pβ value at each residue substitutes for the actual β-sheet propensity and, whether large or small, represents the regional structures of PrP^Sc^. These assumptions allow the use of Pβ graphs as surrogate models for PrP^Sc^ structure.

In this study, we confirmed the biological relevance of Pβ by application to *in vitro* experiments from the literature. Moreover, we incidentally found that the propensity to random coil structure (Pc) values are also important, being apparently related to the cross-seeding efficiencies of amyloidogenic peptides. We subsequently reviewed the results of interspecies transmission experiments reported in the literature from the viewpoint of the above assumptions. Pβ analysis visually aided the interpretation of experimental transmission involving heterologous PrP molecules, as demonstrated by explanation of the differences in incubation times among different hamster species and the changes in host range after interspecies transmission.

## Results and discussion

### Pβ-graphs of representative species

**Fig 1:**
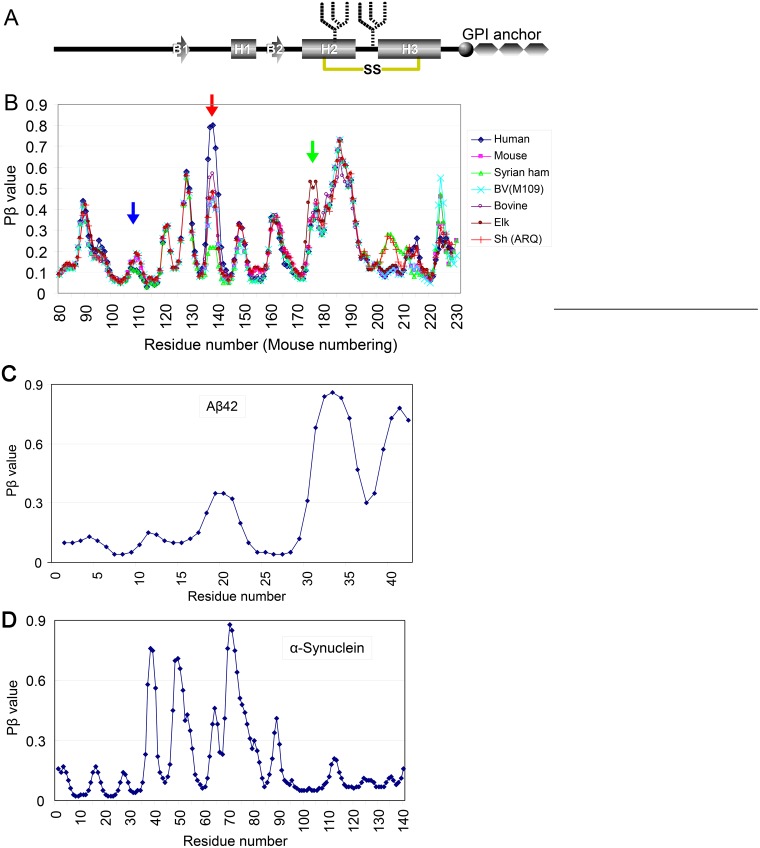
Pβ graphs of PrP of representative species look very alike but with certain differences. **A**. Schematic illustration of the secondary-structure components of prion protein (PrP) and post-translational modifications, including disulfide links (yellow line with "SS"), N-linked glycans (fork-like objects) and glycosylphosphatidylinositol anchor (GPI anchor). B1 and B2, the first and the second β-strands, respectively. H1, H2 and H3, the first, second and third α-helices, respectively. **B**. Pβ graphs of PrP from various species. The blue, red and green arrows indicate peaks centered at residues 110, 140 and 175, respectively. Syrian ham, Syrian hamster; BV(109M), bank vole with methionine at residue 109 (in bank-vole numbering); Sh(ARQ), sheep with the A136/R150/Q171 polymorphism. **C and D**. Pβ-graphs of Aβ42 and human α-synuclein, respectively.

In comparing Pβ graphs of PrPs from representative species, we found them mostly the same as expected from the high homology of the primary structures, except for variations in the heights of some peaks (Fig 1B). For example, Syrian hamster PrP and human PrP had a relatively low peak centered at residue 110 (Fig 1B, **blue arrow**), whereas human (Fig 1B, **red arrow**) and elk (Fig 1B, **green arrow**) PrPs had much higher peaks centered at residues 140 and 175, respectively. The significance of the differences in Pβ peak heights is described in the following sections. Incidentally, we observed more Pβ peaks in PrP than in Aβ42 and α-synuclein (Fig 1C and 1D). According to the initial assumption, the regions with Pβ peaks would be present in β-sheets in template PrP^Sc^, and the similar structures should be induced in the substrate PrP^C^ through direct contact with the high-Pβ regions functioning as interaction “interfaces” with PrP^Sc^ [12]. Possibly, the existence of many peaks may be responsible for the strain diversity of prions. Whether the Pβ-peaks actually correspond to regions with high affinity for PrP^Sc^, and are therefore candidates for interfaces, was critical for this purpose. We therefore attempted to assess the biological relevance of Pβ values.

### Testing biological relevance: Application to ΔPrP-series and C-terminally-truncated mutant PrP

**Fig 2:**
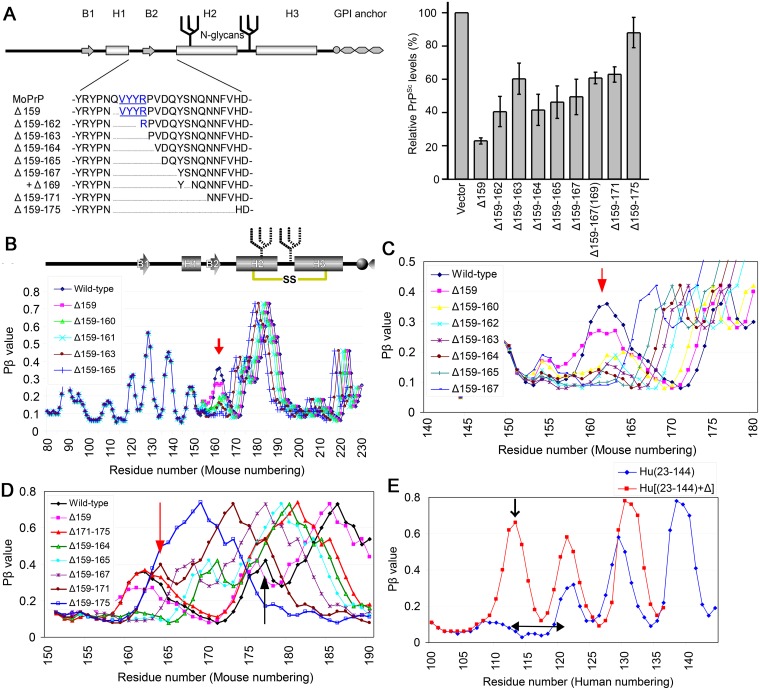
Biological relevance of Pβ: heights of Pβ peaks correlate with dominant-negative inhibition of ΔPrP-series mutants. **A**. (**Left panel**) Schematic illustration of mutant PrP with deletions in the region between H1 and H2 (ΔPrP-series); (**right panel**), graph showing the PrP^Sc^ levels of convertible PrP coexisting with the indicated non-convertible ΔPrP mutant on 22L-infected N2a cells (adapted from reference [13]). The PrP^Sc^ levels of convertible PrP increased as the size of the deletion of the co-existing ΔPrP increased, indicating that the dominant-negative inhibitory effects of non-convertible ΔPrP on co-existing convertible PrP are decreased as the size of the deletion increased. MoPrP, wild-type mouse PrP. **B**. Pβ graphs of wild-type mouse PrP and ΔPrP mutants. The height of the peak around residue 160 gradually decreased as the size of the deletion increased (red arrow), but had little effect on the appearances of the rest of the Pβ graphs. **C**. Pβ graphs of wild-type mouse PrP and ΔPrP mutants, focusing on the region from residue 140 to residue 180, showing the reduction of the peak more clearly. **D**. Pβ graphs of wild-type mouse PrP and ΔPrP mutants, focusing on the region from residue 150 to 190, specifically on the peak around residue 175 (black arrow). The peak became narrower in Δ159–171 (red arrow) and finally disappeared in Δ159–175 (blue curve with open squares). **E**. Pβ graphs of C-terminally-truncated human PrP (Hu23–144) and a deletion variant of the truncated mutant Hu23–144+Δ, with an internal deletion of residues 113–120 (left-right arrow) [14]. The latter protein contained another large peak (arrow).

To test the biological relevance of the Pβ graphs, we applied the algorithm to a series of mutant PrPs with various internal deletions from residues 159–175 (ΔPrP-series, Fig 2A, **left panel**) [13]. An inverse correlation between the sizes of the deletions and the efficiency of dominant-negative inhibition (DNI) of the deletion mutants (Fig 2A, **right panel**) indicated that this region is the main interface by which ΔPrP binds the template PrP^Sc^. The Pβ graph of wild-type mouse PrP showed two peaks, or interface candidates, in the region of residues 159–175 (Fig 2B). As the size of the deletion was increased, from residue 159 toward the C-terminus, the Pβ peak centered around residue 160 was gradually reduced in height (Fig 2B or 2C, **red arrow**), with the peak almost disappearing in Δ159–166. The other peak centered at around residue 175 (Fig 2D, **black arrow**) also became narrower in Δ159–171 (Fig 2D, **red arrow**) and finally disappeared in Δ159–175 (Fig 2D, **blue line with open square**). Collectively, the gradual disappearances of these two peaks of the Pβ graphs of the ΔPrP series correlated with the gradual decrease of DNI of the mutants [13]. We previously described a presumed mechanism of DNI by non-convertible mutant PrP molecules [12]. Briefly, there are two types of non-convertible mutant PrP molecules: one with a functional interface for interactions with the template PrP^Sc^ and the other with a defective interface. The former can bind and occupy the template PrP^Sc^ to inhibit interactions of coexisting convertible PrP, analogous to competitive inhibitors of enzymes. On the other hand, the latter cannot inhibit due to its inability to bind the PrP^Sc^. The loss of two peaks with the concomitant diminution of DNI can be best explained by an assumption that the region represented by the two Pβ-peaks lost by the deletions constitute an interface for the interactions with the template PrP^Sc^. In conclusion, the correlation of these alterations in Pβ graphs with the actual biological events provided evidence for the biological relevance of the values generated by the algorithm.

We also applied the algorithm to the C-terminally-truncated mutant human PrP, PrP23–144. A deletion of residues 113–120, which were thought to constitute a β-sheet core of the amyloid, did not affect the amyloidogenicity of the protein, to their surprise, with solid-state nuclear magnetic resonance (NMR) of the variant showing an altered β-core encompassing resides 106–125 [14]. Pβ graphs of PrP23–144 and the deletion variant (Fig 2E) showed that the internal deletion Δ113–120 generated a large peak, although its exact position differed from that shown by solid-state NMR. This suggested that Pβ analysis could have predicted the newly-formed β-sheet core.

### Testing biological relevance: Significance of Pβ and Pc values on cross-seeding efficiencies

**Fig 3:**
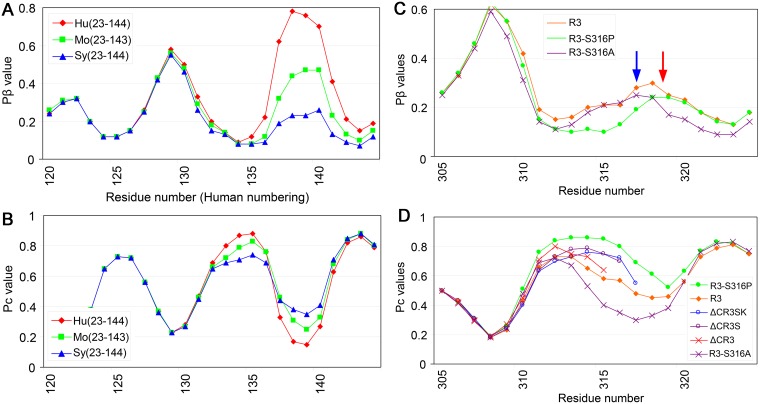
Smaller discrepancies in Pc values in Pβ trough regions are advantageous for cross-seeding of amyloids. **A**. Pβ graphs of Hu23–144 and the equivalent C-terminally-truncated mouse (Mo12–143) and Syrian hamster (Sy23–144) PrPs [15]. Hu23–140 had the highest peak at residue ~140, while Sy23–144 had the lowest and Mo23–143 had an intermediate peak. Peak heights correlated with amyloid formation efficiency, with Hu23–144 being the most efficient, Sy23–144 being the least efficient and Mo23–143 being intermediate. **B**. Pc graphs of Hu23–144, Mo23–143 and Sy23–144. The Pc peak at residue ~135 was located in a Pβ-trough at a position between two Pβ-peaks (compare with **Fig 3A**). The Pc peak is highest in Hu(23–144), lowest in Sy(23–144) and intermediate in Mo(23–143). **C**. Pβ graphs of tau-derived amyloidogenic peptide, R3, and its substitution variants, R3-S316A and R3-S316P, whose serine residue at the codon 316 was replaced with alanine or proline, respectively [16]. The two variants had Pβ peaks at different positions (red and blue arrows). The wide Pβ trough of R3-S316P might explain the inefficiency of fibril formation owing to the overly high freedom of motion in the loop/kink between the two strands. **D**. Pc graphs of R3, the two substitution variants, and C-terminally-truncated variants, ΔCR3SK, ΔCR3S and ΔCR3. ΔCR3S and ΔCR3SK have serine residues at codon 316, whereas ΔCR3 lacks this codon. The curves of ΔCR3S and ΔCR3SK were similar to those of R3-S316P, whereas the curve of ΔCR3 was closer to the wild-type R3. R3-S316A is rather far from R3-S316P.

In evaluating this type of truncated mutant, we were interested in experiments of cross-seeding reactions among PrP23–144 from human (Hu23–144), mouse (Mo23–143) and Syrian hamster (Sy23–144) [15]. Hu23–144 could be cross-seeded with *in vitro*-generated fibrils of Mo23–143 but not Sy23–144, whereas Mo23–143 was cross-seeded by Hu23–144 and Sy23–144 fibrils. In contrast, neither human nor mouse fibrils could seed Sy23–144. Pβ graphs of the C-terminal regions of these molecules clearly differed, with human being highest, hamster lowest and mouse intermediate (Fig 3A); these differences correlated with their fibril-formation efficiency [15]. Using the same algorithm, we assessed the theoretical propensity to random-coil structure (Pc) of these molecules, finding unequivocal differences among the three in the region of residues 132–136, which forms a peak in the Pc graph (Fig 3B) and a trough in the Pβ graph (Fig 3A). Because these findings suggested that Pc values may significantly affect cross-seeding efficiency, we reviewed a series of cross-seeding experiments of an amyloidogenic peptide derived from tau protein (R3) and its variants, e.g. C-terminally-truncated variants and substitution variants with the serine at residue 316 replaced by either proline (R3-S316P) or alanine (R3-S316A) [16]. Experiments showed that fibril formation by R3-316P alone was very slow but could be enhanced by adding fibrils of wild-type R3 or other variants as a seed. However, R3-S316A and C-terminally-truncated variants which lack a serine at residue 316 did not have seeding effects. Pβ graphs showed that the peptides R3-S316A and R3-S316P had peaks at different positions (Fig 3C, **blue and red arrows**). On the Pc graphs, R3-S316P and the C-terminally-truncated variants ΔCR3SK and ΔCR3S, which could seed R3-S316P, showed similar curves, whereas another truncated variant ΔCR3, which could not seed, showed a pattern closer to that of wild-type R3 (Fig 3D). The possible relationship between seeding effects and Pc is discussed in detail below.

### Testing biological relevance: Effects of a prion-resistant polymorphism

**Fig 4:**
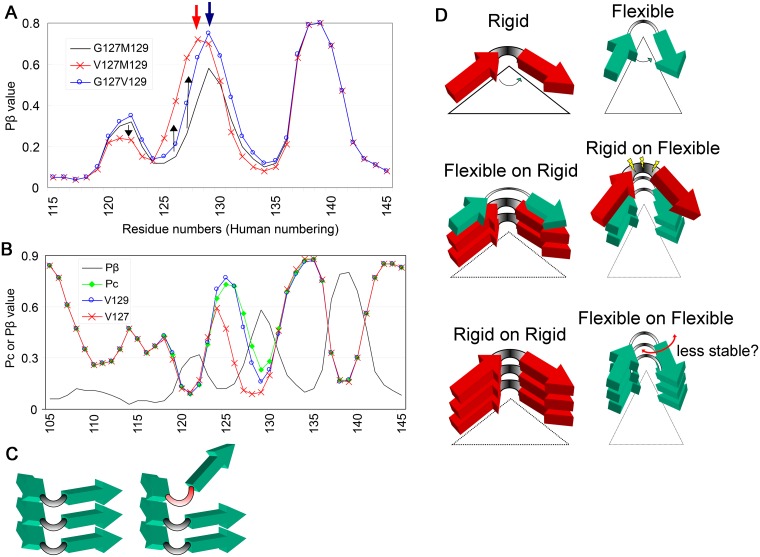
Positions of the β-strands and flexibility of the loop/kink region may affect cross-seeding efficiencies. **A**. Anti-prion effects of a polymorphism V127 may be attributable to alterations in Pβ and Pc. Comparison of Pβ graphs of human wild-type PrP (G127M129) with PrPs with the polymorphisms V127 (V127M129) or V129 (G127V129). The red and blue arrows indicate the crests of peaks of V127M129 and G127V129, respectively. Black arrows indicate differences in Pβ values of V127M129 from those of wild-type. **B**. Pc graphs of wild-type human PrP and PrPs with the polymorphisms V129 and V127. The gray curve represents the Pβ graph of wild-type human PrP. **C**. Schematic illustration of the possible effect of the loop/kink region on parallel in-register β-sheet formation. Differences in the properties of loop/kink regions, such as flexibility or length, would result in the inability of these peptides to form stable parallel-in-register β-sheets. **D**. Schematic illustrations of two types of β-loop-β motifs (β-arch) with either a rigid (upper panels, red arrows, thick lines) or a flexible (green arrows; thin lines) loop. The middle panels illustrate heterologous template-substrate conversion reactions: the left panel shows a reaction in which the template has rigid loop/kink regions and the substrate has a flexible loop, whereas the right panel shows the opposite case. The bottom panels illustrate homologous substrate-template conversion reactions.

We compared a conversion-resistant polymorphism of human PrP, valine at residue 127 (V127) [17], with another polymorphism, having valine at residue 129 (V129). V127 showed a shift of the Pβ peak at residue 129 (Fig 4A, **red arrow**) with elevation of the residues 125–128 region (**black upward arrows**) and reduction of the peak at residue 122 (**black down-ward arrow**). In contrast, V129 simply showed an elevated peak at residue 129, despite their being only one residue apart (Fig 4A, **blue arrow**). A comparison of the Pc-graphs of V129 and V127 (Fig 4B) revealed that the Pc peak of V127 in the Pβ trough region (**gray curve**) was lower and narrower than that of wild-type or V129. Remodeling of Pβ and Pc by V127 may be responsible for its resistance to conversion to PrP^Sc^, because a PrP with a shifted β-strand would have difficulty in aligning side-by-side with its counterpart of the template PrP^Sc^ to form stable parallel in-register β-sheets. A similar explanation would also apply to the tau-derived peptides R3-S316A and R3-S316P, which had Pβ peaks at different positions and did not cross-seed (Fig 3A). In addition, properties of the loop/kink regions connecting the β-strands may have affected parallel in-register β-sheet formation (Fig 4C). Hennetin and colleagues suggested significance of β strand-loop-β strand motifs (β-arch), especially of the loop region (β-arc), for parallel in-register β-sheet formation [18]. Their algorithm ArchCandy was found to predict the amyloidogenicity of some representative amyloids and even structures of the β-arches [19]. Although ArchCandy failed to explain the results of experiments on PrP23–144, the β-arc theory is both reasonable and promising. If Pc values also represent the structures of the β-arcs, then the β-arc theory could explain differences in cross-seeding efficiencies of PrP23–144 and tau R3-derived peptides; i.e. peptides with similar Pc values in the Pβ trough can cross-seed because they have similar β-arcs. Alternatively, differences in cross-seeding efficiencies may be due to the greater adaptability of a flexible than a rigid loop/kink of the substrate peptide, allowing β-strands to be arranged in appropriate positions and orientations for incorporation into the template, particularly in heterologous reactions (Fig 4D, **middle left panel**). Thus, differences in cross-seeding efficiencies of human, mouse and Syrian hamster PrP23–144 may be due to differences between flexible and rigid loop/kink structures. That is, Sy23–144, with the lowest Pc, has a rigid loop/kink, is least adaptive and cannot be cross-seeded. In contrast, Mo23–144 and Hu23–144 have more flexible, adaptive loop/kink structures, making them more prone to cross-seeding in heterologous reactions. In homologous reactions, less flexible loop/kink structures may be more advantageous in parallel in-register β-sheet formation because the restricted freedom of motion would enhance arrangements of β-strands (Fig 4D, **bottom left panel**). To more quantitatively evaluate the biological relevance of Pβ and Pc, we used fluorescence intensity data on green fluorescent protein (GFP) fused to Aβ42 mutants [20] [S1 Fig and S1 Text (Results and Discussion)]. Briefly, although Pβ alone showed a fair correlation with fluorescence intensity which is the indicator of the aggregation tendency (correlation coefficient -0.768) (S1B Fig), the ratio of Pβ and Pc exhibited an even better correlation (correlation coefficient -0.833), supporting our view (S1C Fig). It should be noted that the view does not necessarily indicate that the parallel in-register β-sheet model is correct, because β-arches are also important structural components of β-solenoids [18]. β-solenoid formation seems similar to a cross-seeding of amyloids in terms of heterologous stacking of two (or more) amyloidogenic peptides, as if “intra-molecular cross-seeding”. Indeed, β-solenoids are valuable examples of physiological parallel β-sheet structures so that Hennetin and colleagues utilized them to classify β-arches [18]. Therefore, it is conceivable that the determinants of cross-seeding efficiency of two parallel in-register amyloidogenic peptides, i.e. the properties and positions of β-strands and loops, would also influence compatibility between the stacking partners in β-solenoids and formation efficiency. In the case, significance of the relative ΔPβ parameter would be also maintained, because it represents deviation from the ideal stacking partners for solenoid formation. Although it is much easier to picture conversion process of parallel in-register amyloids than that of the β-solenoid [7], further evidences are required to determine which is more plausible. Either way, we believe that our suggestion would contribute to understanding of mechanisms of certain problems about prion.

### Versatility of Pβ in interpretation of transmission results: Introduction of relative ΔPβ

**Fig 5:**
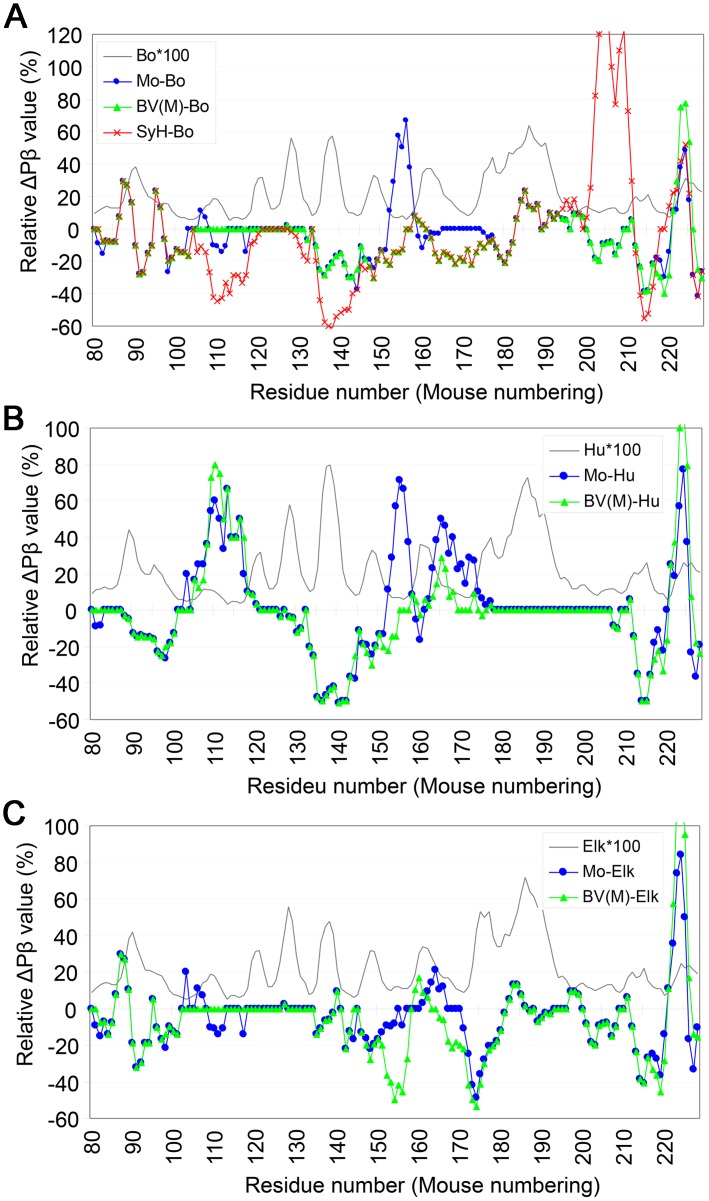
Relative ΔPβ graphs can reveal subtle differences in Pβ among PrPs from different species. **A**. ΔPβ graphs of PrP from mouse (Mo), Syrian hamster (SyH) and bank vole with methionine at the polymorphic codon 109 [BV(M)] relative to bovine PrP (Bo). The gray curve without any marker represents a Pβ graph of bovine PrP, multiplied by 100 to enable comparisons with the ΔPβ-graphs (Bo*100). **B and C**. Comparisons of ΔPβ-graphs of mouse and bank vole with M109 relative to human PrP (**B**) and elk PrP (**C**). The relative ΔPβ-graph patterns differed substantially depending on the species used for reference.

Because Pβ appeared to be biologically relevant, we assessed the versatility of numerical conversion of the primary structure of PrP by analyzing the results of interspecies transmission experiments. However, variations in peak heights made Pβ graphs inconvenient for comparisons of PrP from different species; for example, it obscured the significance of differences in Pβ values in regions with relatively low Pβ values. Because PrP^Sc^ already has β-sheets in these regions, irrespective of their theoretical Pβ values, and because refolding of the substrate PrP^C^ is assisted by the β-sheet-rich PrP^Sc^ in a template-guided manner, we hypothesized that a parameter relative to the absolute Pβ values of the template PrP would better represent the impacts of differences in the primary structures on the PrP^C^-PrP^Sc^ conversion. Therefore, we used a parameter, relative ΔPβ, which represents deviations in Pβ between the substrate PrP^C^ and the template PrP^Sc^. We applied this new parameter to PrP from three intensively investigated species, mouse, bank vole and Syrian hamster, and found it useful for comparisons among different species (Fig 5A). Mouse and bank vole PrPs differed especially in the region of residues 150–175, a region that includes the two residues, 154 and 169, reported responsible for the unique transmission properties of bank vole PrP [21]. As expected, the ΔPβ graph patterns differed greatly depending on the reference species; e.g. human (Fig 5B) or elk (Fig 5C).

Incidentally, we applied this parameter to comparison of representative prion-resistant species (S1 Text and S2 Fig) and found relative ΔPβ rather useful because it accentuates subtle differences on Pβ.

### Interspecies transmissions between various hamsters

**Fig 6:**
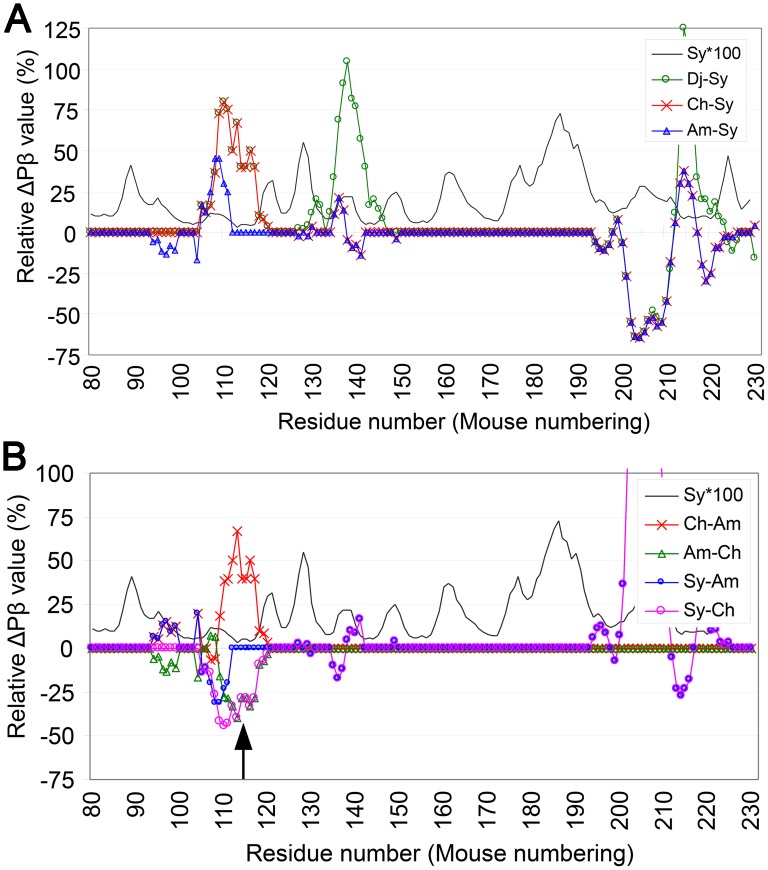
Relative ΔPβ graphs of transmissions of Sc237 among various hamster species visually aid interpretation of the results. **A**. ΔPβ graphs of PrPs of various hamster species relative to that of Syrian hamster for the transmission of Sc237 from Syrian to other hamster species. The reference species are the donors of the transmissions and the former species are the recipients. Sy, Syrian hamster. Ch, Chinese hamster. Am, Armenian hamster. Dj, Djungarian hamster. **B**. ΔPβ graphs for back-transmissions of Sc237 from different hamster species to Syrian hamsters, and for transmission between Armenian and Chinese hamsters. The upward arrow indicates the position of the Pβ trough at residue ~115.

The interspecies transmission efficiency of a prion is thought to depend on whether the conformation of the inoculated PrP^Sc^ is included within the repertoire of conformations that can be adopted by the PrP of the recipient species [22]. Because graphs of relative ΔPβ can indicate deviations in Pβ between two PrPs, we hypothesized that these graphs would reflect differences in the repertoire of conformations between substrate and template PrPs. We analyzed the experimental transmission of the Syrian hamster prion Sc237 to Chinese hamsters and Armenian hamsters [23] and the transmission of Syrian hamster prion 263K, which is regarded to be the same as Sc237, to a similar set of hamster species [24]. Inoculation of Sc237 into Armenian and Chinese hamsters resulted in disease development after incubation periods of ~174 and ~344 days, respectively [23]. As the primary structures of PrPs of Armenian and Chinese hamsters differ at three residues in a narrow region, 102, 107 and 111, the differences in incubation periods were likely due to these residues. ΔPβ graphs relative to Syrian hamster (Fig 6A) showed that differences in primary structures were reflected as distinct sizes of positive peaks in the region of residues 95–120, with positive ΔPβ peaks centered at residue 110 apparently correlating with lengths of incubation; the higher and wider the peak is, the longer incubation periods Sc237 requires. This view is supported by the long incubation period of ~314 days observed in the transmission of Armenian hamster-passaged Sc237 to Chinese hamster, in which the graph of relative ΔPβ showed a large positive peak in the region of residues 110–120 (Fig 6B, **red curve**). The aversion to high Pβ values in this region by this particular strain Sc237 exemplifies that high Pβ value is not always advantageous for efficient transmission. Interestingly, the incubation periods during back-transmissions of Chinese hamster- and Armenian hamster-passaged Sc237 to Syrian hamsters were both similar and relatively short, being ~121 and ~113 days, respectively. Although these phenomena were regarded as evidence for host factors to determine incubation length [23, 24], they may also have resulted from the effects of loop/kink structures on PrP^C^-PrP^Sc^ conversion. The relative ΔPβ graphs of the forward and back transmissions were almost mirror images of each other as expected from the formula, with the positive peaks in the former being negative peaks in the latter and *vice versa* (Fig 6B). In the transmission from Chinese hamster to Syrian hamster, a substantial part of the negative peak in the residue 105–120 region matched the Pβ trough (Fig 6B, **arrow**). If a Pβ trough represents a loop/kink, as discussed above, a lower Pβ in a trough could represent a more flexible loop/kink and be more adaptive to a heterologous template (Fig 4D), reasons that may explain the relatively small difference in incubation periods. Notably, Djungarian hamsters showed relatively short incubation periods for Syrian hamster 263K, despite having the same ΔPβ graph pattern as Chinese hamsters in the region of residues 95–120 (Fig 6A, **green curve**), as if the negative effects of the positive peak at residue 110 were neutralized by another positive peak at residue ~140. This may have been due to the stronger interactions of Djungarian hamster PrP with the template PrP^Sc^ through the interface at residue ~140 resulting from the higher Pβ. These strong interactions may keep the substrate and template PrP molecules close for a time sufficient for the completion of structural changes at the other interface region at residue ~110. Another possibility is discussed below.

### Changes of host range of C-BSE through interspecies transmission

**Fig 7:**
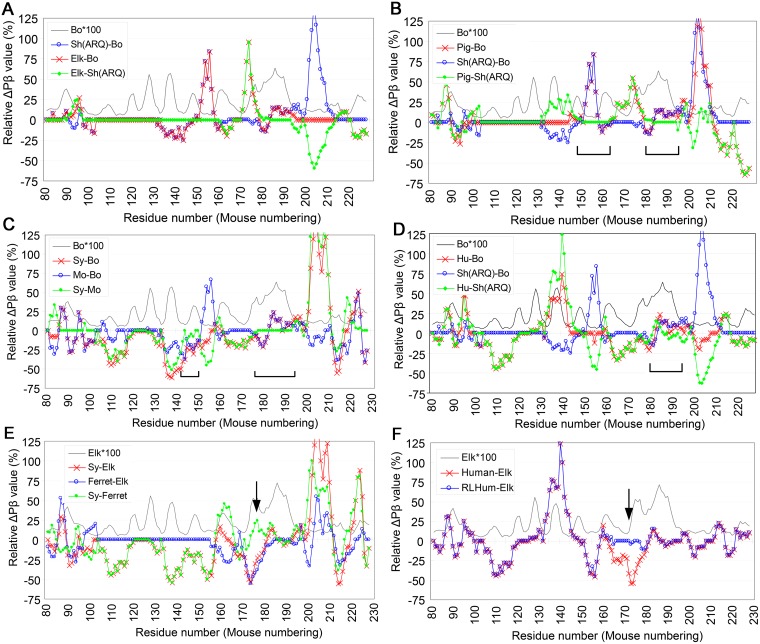
Relative ΔPβ graphs of interspecies transmissions visually aid interpretation and are suggestive of how changes of host ranges could occur. **A**. Relative ΔPβ graphs of transmissions of C-BSE from cattle to sheep with the ARQ polymorphism [Sh(ARQ)-Bo] and from cattle to Tg mice expressing elk PrP (Elk-Bo), and transmission of ovine PrP-adapted C-BSE to Tg mice expressing elk PrP [Elk-Sh(ARQ)][26]. The latter species are donors and the former species are recipients of transmissions. The blue, red and green curves represent efficient, inefficient and improved transmissions, respectively. **B**. Relative ΔPβ graphs of transmissions of C-BSE from cattle to Tg mice expressing porcine PrP (Pig-Bo) and of ovine PrP-adapted C-BSE to Tg mice expressing porcine PrP [Pig-Sh(ARQ)]. The bottom brackets indicate regions flattened by passage through sheep [27]. **C**. Relative ΔPβ graphs of transmissions of C-BSE from cattle to mouse (Mo-Bo) and Syrian hamster (Sy-Bo) and from mouse-adapted C-BSE to Syrian hamster (Sy-Mo) [28]. **D**. Relative ΔPβ graphs of transmissions of C-BSE from cattle to Tg mice expressing human PrP (Hu-Bo) and of ovine PrP-adapted C-BSE to human [Hu-Sh(ARQ)] [25]. **E**. Relative ΔPβ graphs of transmissions of CWD from elk to ferret (Ferret-Elk) and to Syrian hamster (Sy-Elk) and from ferret-adapted CWD to Syrian hamster (Sy-Ferret). The large negative peak at around residue 175 disappeared after passage through ferret (arrow) [29]. **F**. Relative ΔPβ graphs of transmissions of CWD from elk to Tg mice expressing human PrP (Human-Elk) and to Tg mice expressing human PrP with elk residues in the region of residues 166–174 (RLHuman-Elk) [31]. The arrow indicates the region in which the graph pattern was changed by substitutions.

TSEs often show drastic changes in transmission properties after interspecies transmission. For example, sheep-passaged C-BSE can be more efficiently transmitted to transgenic (Tg) mice expressing human [25], elk [26] and porcine [27] PrP than cattle C-BSE. In addition, mouse-passaged C-BSE can be transmitted to Syrian hamsters [28], and ferret-adapted CWD can more efficiently infect Syrian hamsters [29]. Besides enhancing virulence, transmission may result in loss of transmissibility to the original species, as shown by the poor transmission of the CWD passaged in bank vole-PrP-expressing Tg mice to elk PrP-expressing Tg mice [30]. We expected that relative ΔPβ analysis could visually aid in the interpretation of the results of these interspecies transmissions and provide a clue to their underlying mechanisms. The relative ΔPβ graph for the transmission of sheep-passaged C-BSE to Tg mice expressing elk PrP (Fig 7A, **green curve**) showed less deviation from baseline than the ΔPβ graph of the transmission of bovine C-BSE to elk (Fig 7A, **red curve**), suggesting that the enhanced transmissibility may have been due to improvements in deviations in Pβ between the template PrP^Sc^ and the substrate PrP^C^. Relative ΔPβ graphs of the serial transmission of C-BSE from cattle to sheep to pigs also showed improvement in deviations in the regions of residues 150–165 and 180–195 (Fig 7B, **bottom brackets**). Improvements in deviations between template PrP^Sc^ and substrate PrP^C^ in these regions may compensate for any remaining deviations in the intervening region. A similar hypothesis may also explain findings resulting from the serial transmission of C-BSE from cattle to mice to Syrian hamsters. That is, the ΔPβ graph is flattened in the region of residues 175–195 and the deviation in the region of residues 145–150 is also improved near the baseline (Fig 7C, **bottom brackets**).

Unlike the foregoing cases, the relative ΔPβ graph for the transmission of sheep-adapted BSE to Tg mice expressing human PrP did not become flat in the region of residues 180–195 but the deviation in sheep-to-human transmission seemed improved compared with that of cattle-to-human transmission (Fig 7D). Collectively, those results imply that improvements in deviations in the regions of residues 150–165 and 180–195 may facilitate the transmissions of C-BSE to originally less-susceptible species. If this implication is proven by other types of methods, it strongly supports the legitimacy of our approach.

### Changes of host range of CWD through interspecies transmission

The relative ΔPβ graph for transmission of ferret-passaged CWD to Syrian hamsters (Fig 7E, **green curve**) showed a new positive peak in the region of residues 170–180 (Fig 7E, **arrow**), a position similar to the characteristically-high Pβ peak of elk PrP at ~175, when compared with transmission of elk CWD to Syrian hamster (**red curve**). These findings were similar to those of a ΔPβ graph showing the transmission of CWD to Tg mice expressing chimeric human PrP with elk residues in the region of residues 166–174 [31]. The chimerization resulted in greater transmission of CWD than observed in Tg mice expressing pure human PrP [31]. On the relative ΔPβ graph, the negative peak in the region of residues 160–180 observed in the ΔPβ graph of elk-to-human transmission was completely buried in the chimeric PrP (Fig 7F, **arrow**). These findings suggested that smaller discrepancies in Pβ values in the region between substrate and template are advantageous for propagation of CWD or CWD-derived prions.

### A possible mechanism of strain diversity

Relative ΔPβ is unambiguously defined by the primary structures of the PrPs of the donor and recipient species. However, even between the same pair of species, some prion strains are transmissible, whereas others are not. For example, cattle C-BSE is transmissible to mouse, whereas L-BSE is not. This could result from strain-specific patterns of usage and/or the predominance of certain interfaces among the multiple interfaces of PrP [12]: therefore, some strains may favor high Pβ values in a certain interface region, whereas other strains favor low Pβ in the same region. For this viewpoint, structural studies of α-synuclein amyloids provides an example. Bousset and colleagues reported that “fibril-type” and “ribbon-type” amyloids of α-synuclein have distinct patterns of β-sheet distribution [32]: The N-terminal part of the ribbon-type formed rigid β-sheets, whereas the same region of the fibril-type was largely disordered, except for one β-sheet spanning 16–20. On the other hand, the ribbon-type did not have β-sheets in a range from 44 to 57 but the fibril-type continually had β-sheets from 38 to 92. Similar variations in distribution of β-sheets may underlie the strain diversity of prion as well, which presumably arise due to stochastic events, environments or presence of cofactors during the initial nucleation of PrP^Sc^ and the traits are subsequently inherited by the offsprings.

Such variations could presumably stem from stochastic events or from the presence of cofactors and/or environments during the initial nucleation of PrP^Sc^, with the traits being inherited by offspring.

### Future problems to be addressed: Intrinsic properties

In addition to Pβ values, other attributes of β-strands of interface regions can theoretically affect interaction efficiencies between a substrate PrP^C^ and a template PrP^Sc^. These include interactions through side chains, such as steric zippers or electrostatic interactions, the twist/torsion of β-strands, and positioning and orientation relative to other strands [33]. These factors may be addressed by molecular dynamic simulation of parallel-in-register amyloids. Alternatively, an algorithm that combines the characteristics of ArchCandy and secondary-structure prediction may be also feasible because both depend solely on the primary structure for calculations. Such an algorithm may provide insights into other aspects of the intrinsic properties of PrPs and further improve the ability to predict regional structures of PrP^Sc^ based on the primary structure. Accurate prediction of the regional structures would enable construction of three-dimensional models of PrP^Sc^ and accurate simulation of PrP^C^-PrP^Sc^ interactions.

### Future problems to be addressed: Extrinsic factors

Although this study demonstrated that Pβ and Pc carry substantial information about the regional structures of amyloids, we are aware that many other factors affect the conversion efficiency of the substrate PrP^C^. These factors, which are incalculable by the algorithm, include post-translational modifications like GPI anchors, N-linked glycans and disulfide bonds between the second and third helices. These disulfide bonds can yield unpredictable results by keeping high-Pβ regions in the vicinity and precipitating their interactions. Even without disulfide bonds, compact folding of PrP^Sc^ as illustrated by the parallel in-register β-sheet model [8], can result in interactions between high-Pβ regions. The above-described relatively short incubation required in the transmission of 263K to Djungarian hamster could have been a manifestation of such effects. Certain types of phospholipids or nucleotides, which have been postulated to be “cofactors” for PrP^C^-PrP^Sc^ conversion, may also affect actual β-sheet propensities [34–36]. These extrinsic factors may greatly contribute to the behavior and strain diversity of prions and should always be considered in investigations involving Pβ and Pc.

By addressing these problems, our approach can lead to the development of new investigative tools that can be applied not only to prions but to other amyloids, such as Aβ and α-synuclein. Advances in understanding of the protein-protein interactions that underlie amyloid propagation and cross-seeding can benefit protein science/engineering as well as the investigation of neurodegenerative diseases.

## Materials & methods

### Primary structures of PrP from various species

Amino acid sequences of PrP of various species were obtained from the UniProt website (http://www.uniprot.org/). These species included mouse (*Mus musculus*), Syrian hamster (*Mesocricetus auratus*), bank vole (*Myodes glareolus*), human (*Homo sapiens*), elk (*Cervus elaphus nelsoni*), cattle (*Bos taurus*), sheep (*Ovis aries*), pig (*Sus scrofa*), Chinese hamster (*Cricetulus griseus*), Armenian hamster (*Cricetulus migratorius*), Djungarian hamster (*Phodopus sungorus*) and ferret (*Mustela putorius furo*). A region from the tryptophan residue of the second-to-last repeat of the octapeptide repeat region (OPR), e.g. residue 80 of mouse PrP, to the putative GPI-attachment site (e.g. residue 230 of mouse PrP) was excised (Table A in S1 File). All residue numbers are those of the mouse unless otherwise noted.

### Secondary structure prediction

The secondary structure of the excised region of PrPs described above was determined using the neural network prediction method [11], available at the website of the Center for Information Biology of Ochanomizu University (http://cib.cf.ocha.ac.jp/bitool/MIX/). For more details of the algorithm, please refer to the supplementary information, S1 Method. Pβ values of each PrP were determined using values of “neural network prediction 2”. These values were used to plot Pβ graphs and to calculate relative ΔPβ values.

### Definition of relative ΔPβ

To determine relative ΔPβ, the selected regions of PrP from different species were arranged so that the proline at residue 101 of mouse PrP and the corresponding proline residues of each other PrP were in the same row of the work sheet, as shown in S1 File. This resulted in most of the corresponding residues of PrP from different species being arranged in the same row.

The relative ΔPβ between PrPs from two species (here A and B) at a given residue was calculated as:

$$\text{Re}latve\;\Delta P\beta_{\text{A-B}}\;=\;\frac{P\beta_{A}\;-\;P\beta_{B}}{P\beta_{B}}\;\times \;100$$

where Pβ_A_ and Pβ_B_ are the Pβ values of the corresponding residues of PrP of species A and B, respectively; in this case, species B is defined as the “reference species”.

### Correlation analysis of Pβ^max^ or Pβ^max^/Pc^max^ with fluorescence intensities of mutant Aβ42

Pβ^max^ is the maximum value of Pβ of a given Aβ42 mutant in the interval from residue 16 to residue 23 (S1A Fig). Similarly, Pc^max^ is the maximum value of Pc in the interval from residue 13 to residue 19. The relative fluorescence intensities were determined based on the lengths of the bars of the graphs of relative fluorescence intensities (presumably representing the mean values) on the paper by De Groot et al. [20] and compared with Pβ^max^ or the ratio Pβ^max^/Pc^max^. The scatter plots and correlation coefficients were generated using functions in Excel2013.

## Supporting information

S1 Fig Pβ values show fair correlation with aggregation efficiencies of substitution-mutant Aβ42 and Pβ^max^/Pc^max^ show even better correlation. **A**. Examples of Pβ and Pc graphs of mutant Aβ42. Pβ^max^, the highest Pβ value in the region of residues 17–23. Pc^max^, the highest Pc value in the region of residues 13–19. The curves labeled with square brackets, e.g. [F19Y], represent the Pc graphs of the mutant, whereas those without square brackets represent Pβ graphs. **B**. Scatter plot showing the correlation between Pβ^max^ values of the mutant Aβ42 and relative fluorescence intensities of Aβ42-GFP fusion proteins. **C**. Scatter plot showing the correlation between the Pβ^max^/Pc^max^ ratio of the mutant Aβ42 and relative fluorescence intensities of the Aβ42-GFP fusion proteins. The correlation coefficient was improved compared with that shown in **S1B Fig**.
(TIF)

S2 Fig Pβ-, Pc- and relative ΔPβ-graphs of PrPs of representative prion-resistant species imply existence of respective ways of resistance. **A**. Pβ-graphs of PrP from representative prion-resistant species, except for mouse. Mouse is presented as an example of prion-susceptible species for comparison. Dog1 and Dog2, canine PrP registered in GenBank as AF022714 and AF042843, respectively. Sh(ARR), sheep PrP with ARR polymorphisms. Note that the peak at ~160 of PrP of horse, dog1 and ARR-sheep are obviously higher than others. On the other hand, pig, rabbit and dog1 have higher peak ~175. **B**. A magnified Pβ-graphs focusing on the peak at ~160. Note The peak of dog2-PrP is slightly shifted to the N-terminal direction (red arrow) and that pig- or rabbit-PrP have higher Pβ values than mouse PrP (blue arrows), making a very small peak at the residue 167. **C**. A ΔPβ-graphs of the resistant species relative to mouse PrP. The differences in Pβ values between species are accentuated. The red and the blue arrows are indicating the same positions as in S2B Fig. **D**. Pc-graphs of the resistant species. Note that dog2-PrP (green line) show lower Pc values than others in the region between the two red arrows. except for dog1-PrP. The color codes of the graphs are same as in **S2B Fig**. **E**. ΔPβ-graphs of the resistant species relative to mouse PrP focusing on the C-terminal region. Dog1-PrP was eliminated, because it is one-residue shorter than others after the residue 191. The color codes of the graphs are same as in S2C Fig.
(TIF)

S1 Text Relations between Pβ and Pc values and aggregation propensity using the data from Aβ42-GFP fusion protein experiments. (DOC)

S1 File Tables A to H, presenting Pβ and Pc values of PrPs from various species, Aβ42 and tau-derived peptides. (XLS)

S1 Method Detailed information about the neural network secondary structure prediction. (DOC)
