# Supplementary figures and images for: Secondary-structure prediction revisited: Theoretical β-sheet propensity and coil propensity represent structures of amyloids and aid in elucidating phenomena involved in interspecies transmission of prions
Source: PLoS One. 2017 Feb 15;12(2):e0171974. doi: 10.1371/journal.pone.0171974 (PMC5310760; doi:10.1371/journal.pone.0171974)

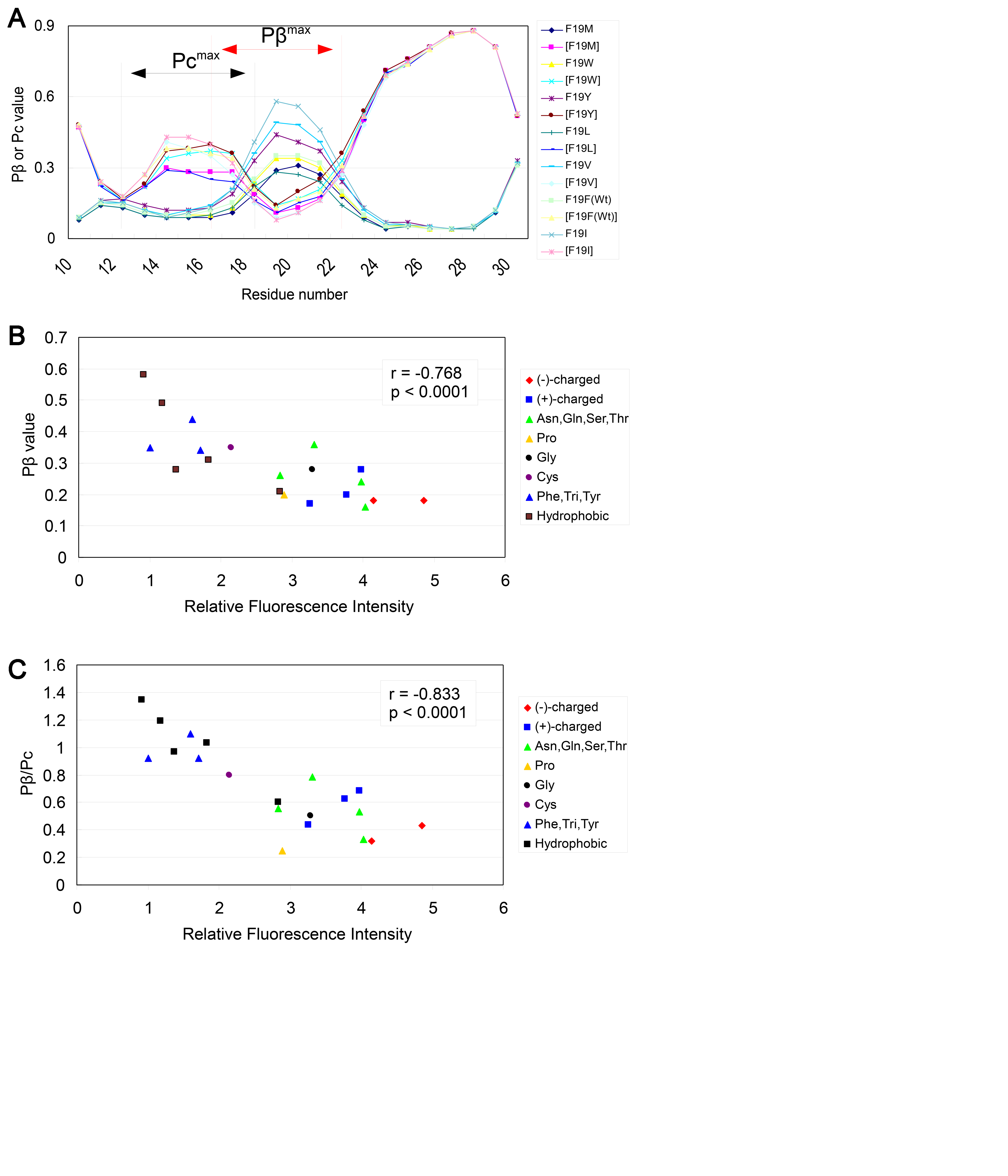

Supplement: S1 Fig — A. Examples of Pβ and Pc graphs of mutant Aβ42. Pβmax, the highest Pβ value in the region of residues 17–23. Pcmax, the highest Pc value in the region of residues 13–19. The curves labeled with square brackets, e.g. [F19Y], represent the Pc graphs of the mutant, whereas those without square brackets represent Pβ graphs. B. Scatter plot showing the correlation between Pβmax values of the mutant Aβ42 and relative fluorescence intensities of Aβ42-GFP fusion proteins. C. Scatter plot showing the correlation between the Pβmax/Pcmax ratio of the mutant Aβ42 and relative fluorescence intensities of the Aβ42-GFP fusion proteins. The correlation coefficient was improved compared with that shown in S1B Fig. (TIF) [file pone.0171974.s001.tif]

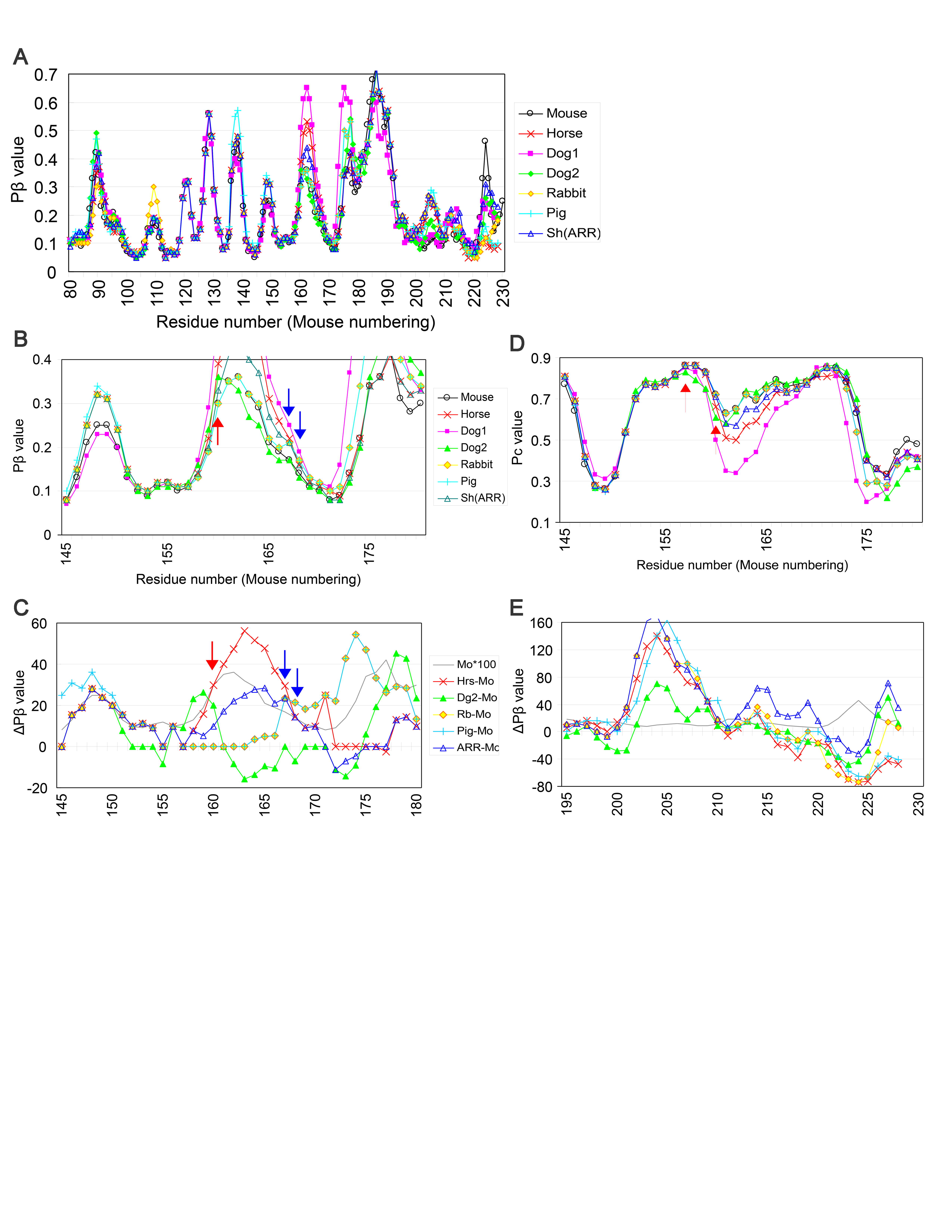

Supplement: S2 Fig — A. Pβ-graphs of PrP from representative prion-resistant species, except for mouse. Mouse is presented as an example of prion-susceptible species for comparison. Dog1 and Dog2, canine PrP registered in GenBank as AF022714 and AF042843, respectively. Sh(ARR), sheep PrP with ARR polymorphisms. Note that the peak at ~160 of PrP of horse, dog1 and ARR-sheep are obviously higher than others. On the other hand, pig, rabbit and dog1 have higher peak ~175. B. A magnified Pβ-graphs focusing on the peak at ~160. Note The peak of dog2-PrP is slightly shifted to the N-terminal direction (red arrow) and that pig- or rabbit-PrP have higher Pβ values than mouse PrP (blue arrows), making a very small peak at the residue 167. C. A ΔPβ-graphs of the resistant species relative to mouse PrP. The differences in Pβ values between species are accentuated. The red and the blue arrows are indicating the same positions as in S2B Fig. D. Pc-graphs of the resistant species. Note that dog2-PrP (green line) show lower Pc values than others in the region between the two red arrows. except for dog1-PrP. The color codes of the graphs are same as in S2B Fig. E. ΔPβ-graphs of the resistant species relative to mouse PrP focusing on the C-terminal region. Dog1-PrP was eliminated, because it is one-residue shorter than others after the residue 191. The color codes of the graphs are same as in S2C Fig. (TIF) [file pone.0171974.s002.tif]
