# Supplementary material for: Secondary-structure prediction revisited: Theoretical β-sheet propensity and coil propensity represent structures of amyloids and aid in elucidating phenomena involved in interspecies transmission of prions
Source: PLoS One. 2017 Feb 15;12(2):e0171974. doi: 10.1371/journal.pone.0171974 (PMC5310760; doi:10.1371/journal.pone.0171974)
Supplement: S1 Text — (DOC) [file pone.0171974.s003.doc]

**S1 Text (Results and Discussion)**

***Relations between Pβ and Pc values and aggregation propensity using the data from Aβ42-GFP fusion protein experiments.***

To further assess the relationship between Pβ values calculated by the secondary structure prediction algorithm and the actual aggregation efficiencies of amyloidogenic proteins, we used fluorescence intensity data of mutant Aβ42-GFP [20]. In those experiments, phenylalanine at residue 19 of the Aβ42 moiety of the fusion protein was replaced by 19 other amino acids and aggregation tendencies of these proteins were assessed by measuring the fluorescence of the GFP moiety. Because most of the alterations in Pβ and Pc occur at a single Pβ peak or a single Pβ trough, this dataset was easy to apply to our system. Comparisons between the maximum Pβ values of the Pβ peaks (**S1 Fig. A**) and the fluorescence intensities revealed a fair degree of correlation, with a correlation coefficient of -0.768 (**S1 Fig. B**). In contrast, we found that Pc in the adjacent Pβ trough region changed in association with fluorescence intensity, although Pc itself did not show substantial correlation with fluorescence intensity. Because we suspected that Pc played a role in amyloid formation, we compared the fluorescence intensities by measuring Pβ^max^/Pc^max^ ratios. Use of this ratio resulted in an even better correlation coefficient, -0.833, than Pβ alone. In contrast, highly-charged residues and polarized residues with relatively large side chains, e.g. glutamine and threonine, remained outliers (**S1 Fig. C**). This improvement suggested that Pc in the adjacent Pβ trough region affected amyloid formation to some extent. Although direct comparisons are difficult, the correlation coefficient was comparable with other amyloid-prediction algorithms [37], suggesting that Pβ and Pc carry substantial structural information on amyloids.

The finding, that many of the outliers are charged residues, suggested that they destabilized amyloid formation by Aβ42 to a greater extent than predicted by the secondary structure algorithm. This was not unexpected, because repulsion between the same electric charges at short distances also occurs in parallel in-register β-sheet amyloids like Aβ42, where the same amino acids are positioned side-by-side.

**Reference**

37. Belli M, Ramazzotti M, Chiti F (2011) Prediction of amyloid aggregation in vivo. EMBO Rep 12: 657–663.

***Pβ and Pc of PrP of prion-resistant species***

Then, we analyzed Pβ, relative ΔPβ and Pc of PrPs from prion-resistant species, namely rabbit (*Oryctolagus cuniculus*), pig (*Sus scrofa*), horse (*Equus caballus*), sheep with ARR polymorphism and dog (*Canis lupus familiaris*), in comparison with mouse PrP (**S2 Fig A**). As for dogs, two canine PrP deposited in GenBank, AF022714 (**dog1**) and AF042843 (**dog2**) are presented. We particularly focused on two regions where PrPs of resistant species seemed substantially different from mouse PrP: one is the region between first and second helices (**H1~H2**) and the other is the region toward the putative GPI-attachment site (C-terminal region). In H1~H2, PrPs of horse, dog1 and ARR-sheep showed obviously higher Pβ-peaks at 162 than that of mouse PrP. Considering that H1~H2 is an important PrP^C^-PrP^Sc^ interaction interface for some prion strains [13], the high Pβ values could contribute to their resistance to prions. Primary structures of pig PrP and rabbit PrP are identical in this region and so were the Pβ-graphs with higher Pβ values than mouse PrP in 167-170, making a small peak at 167 (**S2 Fig B, blue arrows**) and merging to the curve of ARR-sheep. Although biological significance of this small peak is unclear, interestingly pig is relatively susceptible to atypical scrapie [27]. PrP of dog2 also showed a unique pattern in this region: the pinnacle of the peak was slightly shifted to 160, almost merging to the curve of horse PrP (**S2 Fig B, red arrow**). Those characteristics of Pβ values were accentuated on ΔPβ-graphs (**S2 Fig C**). Uniqueness of dog2-PrP was more obvious in the Pc-graph, where Pc values of dog2-PrP were lower than other PrPs, except for dig1-PrP, in a range 157-160 (**S2 Fig D, green line between red arrows**).

Primary structures of the C-terminal region of some resistant species were uniquely varied: Specifically, rabbit PrP, -E_220_SQAAYQRA-; swine PrP, -E_220_YEAYAQRG-; and, equine PrP, -E_220_YEAFQQRG-. Accordingly, their Pβ values were varied as illustrated by the ΔPβ-graphs relative to mouse PrP (**S2 Fig E**). Those unique sequences could also contribute to the resistance of those species, because propagation efficiencies of certain prion strains are highly affected by the primary structures in the region. For example, transmission of elk CWD is affected by the F225 polymorphism (in elk-PrP numbering) [38]. E226 polymorphism influences stability of CWD strains [39]. In transmission experiments of CWD to Tg mice expressing elk-mouse chimeric PrP, mouse residues in the C-terminal region substantially elongated incubation periods [40].

Collectively, Pβ or Pc values of PrPs of the resistant species were rather varied in H1~H2 and C-terminal regions, implying that resistance to prion depends on their respective unique properties of PrP.

**References**

38. Angers R, Christiansen J, Nalls A V, Kang H-E, Hunter N, et al. (2014) Structural effects of PrP polymorphisms on intra- and interspecies prion transmission. Proc Natl Acad Sci U S A 111: 1–6.

39. Angers RC, Kang H-E, Napier D, Browning S, Seward T, et al. (2010) Prion strain mutation determined by prion protein conformational compatibility and primary structure. Science 328: 1154–1158.

40. Tamguney G, Giles K, Oehler A, Johnson NL, DeArmond SJ, et al. (2013) Chimeric elk/mouse prion proteins in transgenic mice. J Gen Virol 94: 443–452.
