# Supplementary material for: Secondary-structure prediction revisited: Theoretical β-sheet propensity and coil propensity represent structures of amyloids and aid in elucidating phenomena involved in interspecies transmission of prions
Source: PLoS One. 2017 Feb 15;12(2):e0171974. doi: 10.1371/journal.pone.0171974 (PMC5310760; doi:10.1371/journal.pone.0171974)
Supplement: S1 Method — (DOC) [file pone.0171974.s005.doc]

**S1 Method**

***The method for secondary structure prediction***

We employed the secondary structure prediction method developed by Ning Qian and Terrence Sejnowski [11]. The algorithm was implemented according to the method described in the paper as is at http://cib.cf.ocha.ac.jp/bitool/NN/. All the parameters for the prediction were taken from Tables 13 to 16 in the paper. The method is based on non-linear neural network models. Three-layer neural network, namely, input, hidden and output layers were employed in the model. The input layer accepts 13 contiguous amino acid residues independently and the weighted information is transferred to the hidden layer. The hidden layer further transferred the weighted information to the output layer. The output layer has three values, each corresponding to the probability of alpha-helix, beta-sheet, and others. The highest value is the predicted secondary structure of the amino acid residue in the center of 13 amino acids. The set of the weights in the network was trained using 106 proteins with known structures to obtain the best prediction performance. Qian and Sejnowski claimed that the success rate of the prediction for test set was 64.3%.

State-of-the-art prediction methods for the secondary structure have achieved the success rate of more than 70% [41, 42]. These high precision methods extract secondary structure information from homologous protein sequences relying on the empirically known fact that homologous proteins maintain their structure. In this study, however, we need to know the impact of a point mutation to the secondary structure, and hence information obtained from the homologous proteins may erase the perturbation introduced by the mutation. We, therefore, used a prediction method that can be applied to and confined to a single amino acid sequence in this study.

**References**

41. Rost B. (2001) Review: Protein secondary structure prediction continues to rise. J Struct Biol 134: 204-218.

42. Garnier J. (1990) Protein structure prediction. Biochimie 72: 513-524.
